# Negro College at Nashville

**Published:** 1884-04-20

**Authors:** 


### *NEGRO COLLEGE AT NASHVILLE.*

The Colored Medical School at Nashville had its eighth commencement on 21st February, and turned out eight graduates. The order of exercises were a little peculiar, as might be expected—first, a salutatory address; second, a valedictory; third, an address to the graduating class; fourth, degrees conferred, and two or three other addresses, all, we suppose, by gentlemen of color, as the account which we have read of the exercises do not state the color of the speakers. The programme was also interspersed with an occasional hallelujah song. The school is called The Mehary Medical College, and has thus far graduated forty-five colored M.D.s.

